# Prerequisites for entry to advanced nurse practitioner studies- a qualitative study of Norwegian nurse anesthetist students’ experiences

**DOI:** 10.1186/s12909-024-05137-3

**Published:** 2024-02-19

**Authors:** Cecilie Aanderud-Larsen, Sara Østlien, Ann-Chatrin Linqvist Leonardsen

**Affiliations:** 1 Sykehuset i Vestfold, Halfdan Wilhelmsens alle 17, 3116 Tønsberg, Norway; 2 Oslo University Hospital, Ullevål, Kirkeveien 166, 0450 Oslo, Norway; 3 University of South-eastern Norway, Raveien 215, 3184 Borre, Norway; 4 Østfold University College, Postal box code 300, 1757 Halden, Norway

**Keywords:** Academic progression, Non-technical skills, Nurse anesthesia, Nurse anesthesia student, Prerequisite, Technical skills

## Abstract

**Background:**

Nurse anesthesia is acknowledged as advanced practice nursing, and requires independency in patient monitoring and clinical decision-making. In Norway, 2022, a prerequisite to nurse anesthesia education programs of at least two years of clinical nursing experience prior to entry, was removed. The consequences of removing the prerequisite of clinical nursing experience prior to entering the nurse anesthetist education program on academic progression or on students’ qualifications after completion of the program remain unexplored. Hence, the purpose of the current study was to explore nurse anesthetist students’ experiences of the impact their previous clinical nursing experience had on their academic progression.

**Methods:**

A qualitative design with semi-structured individual interviews was used. The sample consisted of 12 nurse anesthetist students at the end of the education program. The data were analyzed using thematic analysis in-line with recommendations from Braun & Clarke.

**Results:**

Two main themes with in total six subthemes were identified, namely 1) Experience develops non-technical skills, with subthemes (a) feeling secure in task management, (b) recognizing different situations, (c) understanding my role in teamwork, and 2) Integration of non-technical and technical skills, with subthemes (a) possessing procedural competence, (b) taking responsibility in medication administration, and (c) including a patient-centred approach. Previous clinical experience as a nurse prior to entry to a NA education program had provided a basis of non-technical and technical competencies, that supported further learning and development of advanced level competencies that are needed for NAs.

**Conclusion:**

Non-technical and technical nursing competence represented a solid base for achievement of anesthesia competence within the same areas, also ensuring patient-centred practice. Hence, the change in prerequisites to the NA education program must be followed by evaluations of consequences on students’ academic progress and competence at the end of the program, as well as a possible increased need for supervision throughout.

## Background

In 2021, the International Council of Nurses (ICN) [1] acknowledged Nurse Anesthetists (NAs) as Advanced Practice Nurses (APNs), and recommended education programs at a minimum of masters’ degree level (120 European Credit Transfer and Accumulation System, (ECTS)). To date, NA education programs vary in length and contents globally, directed by national legislations and needs [2]. Despite similarities in NAs’ role and responsibilities in the Nordic countries, there are great variations in education programs, comprising from 30 to 120 ECTS [3]. In Norway, NA education programs previously comprised 90 ECTS [4]. One of the prerequisites for entry to the program was a minimum of two years of relevant, full-time, clinical experience as a nurse. From 2012, masters’ degree programs have been established, and in 2022 a national guideline for NA education programs was implemented [5]. The guideline specifies that all NA education programs must as a minimum include 30 weeks of clinical practice of 30 h each. Prerequisites are having a bachelor degree in nursing, and a Norwegian authorization as a nurse. ICN states that a prerequisite for entry to NA education programs is a minimum of one-year nursing experience, preferably in acute care [1]. However, as a consequence of the national guideline, several Norwegian universities have discarded the prerequisite of nursing experience, allowing for students to enter directly from the nursing bachelor program to the NA education program.

### Summary of existing literature

Only two studies focusing on the impact of clinical nursing experience as a prerequisite to NA education programs have, as far as we know, been conducted the last 12 years. In 2011, Burns [6] identified a statistically significant positive relationship between prerequisites such as grades and critical care experience, and academic progression for NA students. In 2014, Collins & Callahan [7] claimed that prerequisites in general do not ensure positive progression and graduation for NA students. However, the aim of their study was to demonstrate the need for valid clinical evaluation tools.

In Norway, NAs traditionally have been qualified to independently administer general anesthesia for minor operations on otherwise healthy patients, and to work in a team with an anesthesiologist on anesthesia for major operations and patients with more complex illnesses [8]. The aim of the current study was to explore NA students’ experiences of the impact their previous clinical nursing experience had on their academic progression towards the independent role as a NA.

## Materials and methods

### Study design

The study had a qualitative design, using semi-structured, individual interviews to explore NA students’ experiences. A qualitative approach is appropriate when aiming to explore how individuals experience a phenomenon, dependent on their background, interests and interpretation [9]. The study is reported in-line with the Consolidated criteria for reporting qualitative research– COREQ [10].

### Participants

NA students were invited to participate at the end of a 90 ECTS NA education program from one university in Southeastern-Norway, which allows a variation from at least nine hospitals in the region. A purposive sampling strategy was used, selecting information rich cases for in-depth study [9]. Maximum variation sampling was sought [11], focusing on inviting participants of both gender, a range in age, from different geographical areas and with a variation in years and sort of previous working experience as a nurse. The inclusion criteria to participate in the study were NA students who were to complete 90 ECTS of the NA education, with at least to years of clinical experience prior to NA education start.

An open invitation was sent out through the program coordinator at the university. NA students in their third semester were invited to participate. Participants were asked to contact one of the first authors to agree on an interview. Pragmatically, the sample was set to 12 participants. This was assumed purposive, due to the narrow research aim, to ensure sufficient information power [12].

### Interview guide

An interview guide was developed based on several discussions within the research group until consensus was reached (Table 1).

**Table 1:** Semi-structured interview guide*

Question	Follow-ups, probes
In what way do you think your previous clinical experience has affected your learning outcome of the clinical practice periods?	Can you tell me about a concrete situation in clinical practice where your previous experience was valuable for your learning outcome? Can you tell me about a concrete situation in clinical practice where your previous experience was an obstacle for your learning outcome?
Can you remember a situation from clinical practice where your previous experience seemed insufficient?	Please elaborate Can you tell me about a concrete situation in clinical practice where you had to ‘reset’ from old habits?
In what way do you think your previous clinical experience has affected your learning outcome of the theoretical lectures?	Can you tell me about a concrete situation in theoretical lectures where your previous experience was valuable for your learning outcome? Can you tell me about a concrete situation in theoretical lectures where your previous experience was an obstacle for your learning outcome?
In what way do you think your previous clinical experience has affected your learning outcome of the simulation exercises?	Can you tell me about a concrete situation in simulations where your previous experience was valuable for your learning outcome? Can you tell me about a concrete situation in simulations where your previous experience was an obstacle for your learning outcome?
To what extent do you feel that your previous experience has enabled transferring theoretical lectures into clinical relevance?	Please elaborate
Which aspects from your previous clinical experience have been useful as an NA student?	Which aspects from your previous clinical experience have been an obstacle as an NA student?
In your opinion, are there any benefits from not having previous clinical experience prior to partaking the NA education?	
Do you have any final thoughts, or something to add that I haven’t asked?	
*Interviews were conducted in English. These questions represent a translated version of the interview guide.	

The interview guide was piloted in one (female) NA, to ensure that the guide was understandable, logic, and relevant. Some of the questions were shortened, using fewer words, two questions were deleted, and the final question was added to the interview guide.

### Data collection

Due to the Covid-19 pandemic, interviews were conducted remotely, through the safe digital platform Zoom, in the period May-June 2022. The interviews lasted from 25 to 55 min. In the first interview, all authors were present, to validate the interview guide and data collection procedure. Remaining interviews were conducted by the two first authors together (CAL, SØ), one of them being the most active part, and the other one taking notes and posing follow-up questions if needed. Repeatedly during the interviews, the least active author orally summarized the participants’ responses as a validation of the participants’ statements [9]. Interviews (sound only) were digitally recorded and transcribed verbatim by one of the authors respectively (CAL, SØ, ACLL).

### Reflexivity

ACLL is a professor and NA, with nine years of clinical experience as a nurse prior to partaking the NA education, and over ten years of NA experience. She is well experienced with qualitative research methods. CAL and SØ are both NAs with 2.5 and 3 years of experience as nurses respectively, prior to partaking the NA education. The study was conducted as part of CAL and SØ’s masters’ degree. Hence, participants were students at the same program as the two first authors. However, in recruitment of participants, the focus was on avoiding inclusion of students familiar to the authors. Also, the last author contributed throughout with critical reflections and input, both during data collection, analysis, and interpretation of findings.

### Analysis

Thematic, inductive analysis in five steps, in-line with recommendations from Braun & Clarke [13], was used to analyze the data. In step one, the two first authors read and re-read the transcripts to get an impression of the whole, and to familiarize with the data. Then, each transcript was coded inductively, by manually marking central key words (CAL, SØ). The codes were then reviewed by the last author (ACLL). In step two, codes across all interviews were collated, and CAL/SØ searched for aspects that were related, and that could be identified as initial sub-themes. Here, all sub-themes were marked with different colors on paper-sheets. In step three, the codes and sub-themes were then assessed and discussed by all authors, until consensus was reached. In step four, the sub-themes were reviewed, and in step five, the final themes and sub-themes were identified and named through iterative discussions among the three authors until consensus was reached.

### Ethics approval and consent for participation

The study was approved by the Norwegian Center for Research Data (NSD) (Ref. no. 267,130), with regards to confidentiality issues. According to Norwegian legislations, no ethics approval is needed when interviewing healthcare personnel [14]. The study was conducted in-line with the Declaration of Helsinki [15]. Participants gave their written, informed consent to participate, and were informed that they could withdraw the consent at any point without any negative consequences. The data was handled confidentially and anonymously, and it is not possible to recognize individuals in the presentation of results.

## Results

In total 12 NA students participated (two male) in semi-structured interviews. The participants had from 3.5 to 20 years (mean 10 years) of experience as a nurse prior to entry to the NA education program. They were recruited from four different hospitals.

All the participants thought that the NA education would have been even more demanding if they hadn’t had previous experience as a nurse, supporting their learning of new theory and skills. With this as a basis, they did not have to learn basic nursing skills and could focus on the anesthesia-specific theory and practice. Through analysis, two main themes with a total of six subthemes were identified. Table 2 gives and overview of the relation between themes and subthemes.

**Table 2:** Overview of themes and subthemes identified

Themes	Subthemes
Experience develops non-technical skills	a) feeling secure in task management b) recognizing different situations c) understanding my role in teamwork
Integration of non-technical and technical skills	a) possessing procedural competence b) taking responsibility in medication administration c) including patient-centredness

### Experience develops non-technical skills

All participants reflected on that previous nursing experience had developed their non-technical skills within different areas.

### Feeling secure in task management

Several of the participants described being used to meeting unforeseen events as a nurse. Several of the participants emphasized their ability to prioritize and work systematically, seeing the whole picture and continuously being prepared for a «worst case scenario». Some stated that, as a new nurse, it is difficult to focus on several things at the same time. Moreover, being used to a high workload and hectic days was seen as useful when meeting new challenges as a NA. Participant 3 stated that experience developed an ability to focus on the work tasks also in chaotic situations. Participant 9 said: «Having experienced chaotic situations- I think it is an advantage. To avoid contributing to the chaos yourself».

However, entering the new role as a NA student had been challenging. Several of the participants underlined the NA’s independent role, making it essential to feel secure on alternative actions, be able to assess the situation and decide what to do. To several of the participants, this felt overwhelming. But, as stated by participant 1, previous experience made this somewhat less overwhelming: «Even only four or five years of experience will make you more prepared and ready for taking action than having no experience as a nurse at all».

### Recognizing different situations

All the participants referred to ‘tacit knowledge’ as being integrated through their previous experience. This was related to being able to recognize complex symptoms based on clinical observations, and being able to identify deterioration in patients’ condition, without being dependent on medical technical equipment. Participant 8 described this as «being able to identify deterioration before it becomes visible at the monitor». All of the participants linked this skill to having several years of clinical nursing experience. Participant 6 underlined: «It takes years to develop tacit knowledge. You must have seen very ill patients enough times before you can be able to recognize them».

Moreover, several of the participants described how previous clinical experience had provided them with a «bank of experiences», that they could add to during the NA education. For example, participant 1 elaborated:

«You know that if the patient gets nauseous, you think blood pressure decrease instead of just.if you’re nauseous you need Afipran».

### Understanding my role in teamwork

Even if being a NA student was a new role for all of the participants, nine of the participants found previous experience with being part of teams as a nurse as a strength when taking their role in new team constructions. Participant 7 stated:

«From the emergency department, I’m used to gather the team if things flow… It’s the nurse’s role to collect the loose threads. Raising my voice is something I’ve learnt in the emergency department, and this has been useful».

Several of the other participants also emphasized having extensive experience with team communication, and that this had made them used to taking their place in the team. Participant 5 underlined the importance of not being afraid to ask if something is unclear, and to create a dialogue. For example, participant 6 thought this had made her not afraid of making the surgeon aware of changes in the patients’ condition during surgery. Specifically, this experience was found to be useful in the collaboration with the anesthesiologist. Previously, the participants had experienced being exposed to authoritarian physicians and reprisals, especially early in the nursing career. Having learnt how to deal with this was seen as a strength when entering the operating room. Participant 7 illustrated it like this:

«When I was new in the emergency department, I thought it was frightening to call a physician if the patient deteriorated. Now, I’m not afraid to call the anesthesiologist if I’m unsecure».

### Integration of non-technical and technical skills

The participants also referred to having achieved technical skills and competence in various practical tasks also included in NAs’ work, due to their previous experience as a nurse. This enabled them to focus on anesthesia-specific issues, to further develop these skills, and last but not least to combine these skills with a patient-centred approach.

### Possessing procedural competence

All of the participants agreed that the NA education program was based on an assumption that students were able to for example insert peripheral vein catheters (PVC), to prepare and administer medications and to handle critically ill patients. Several of the participants stated that without previous nursing experience, much effort would have been put into learning basic nursing skills and procedures. This would impact the focus on learning anesthesia-specific skills and procedures. Participant 12 said: «You have to train a lot on the basic skills before being able to advance to a higher level». Additionally, several of the participants had experience from specialized hospital wards, and hereby experience with non-invasive ventilation, ventilators and handling of multiple infusion pumps. This was also seen and advantage in the specialized setting the operating room is.

Additionally, this was related to having experience from an high-technological environment, which reduced the need to use time to learn to master the equipment.

### Taking responsibility in medication administration

The participants described that their previous experience had contributed to an understanding of medications and medication administration, that increased their respect for this aspect of nursing. One of the participants [3] also underlined the importance of having administered medications without supervision. Participant 8 said: «No stress for me in the operating room when we needed to add a pressor, when the patient got unstable, or in airway difficulties. I’m used to such issues.» Participant 9 also described having an increased respect for the medications’ potency throughout his career, for example when in retrospect acknowledging having administered potent drugs too fast. He stated: «Many things I’ve learnt is scary when reflecting on it in retrospect. Having sedated patients on propofol, without knowing….I would have been more afraid now than then…So, overdosing, handling respiratory challenges, I have more respect and knowledge now than before».

### Including a patient-centred approach

Most of the participants agreed that having experience with high-technological equipment gave an ability to see beyond the equipment itself, and focus on the patient. They emphasized that due to their previous knowledge and skills, the equipment was almost ‘invisible’. Participant 1 described it like this:

«I am so used to these technical gadgets.The equipment surrounding us, so this doesn’t distract me much. I don’t need to use much energy on it, and can provide safety and focus on the patient».

The balance of vigilance on the patient and on the equipment was described by several of the participants. For example, participant 7 stated:

«I noticed in practice that there are so many screens you’re supposed to overview, so it’s easy to forget the person lying there. But, I think that my previous experience with monitors…the screens…made it easier to week out the screen and rather monitor the patient».

However, one of the participants [12] reported that she did not get enough time to learn the monitoring equipment during the NA education, and that this had led to the patient being ignored.

## Discussion

This is, to our knowledge, the only study focusing on students’ experiences with clinical experience as a prerequisite to NA education. Our results indicate that previous clinical experience as a nurse prior to entry to a NA education program provides a basis of non-technical and technical competencies, that supports further learning and development of advanced level competencies that are needed for NAs.

In the current study, all participants stated that their previous nursing experience had developed their non-technical skills relating to task management, recognizing and interpreting various situations, and taking part in a new role, as a NA, in teamwork. These competencies are in-line with the non-technical skill areas described by e.g. Flynn et al. [16], namely situation awareness (recognizing and understanding), decision making (assessing risks and selecting options, re-evaluating), task management (prioritizing, planning and preparing), and teamworking (displaying authority and assertiveness, assessing roles and capabilities) [17]. Clinical decision-making is essential in anesthesia, and has been described as a central nursing practice task that links a nurse’s perceptions with behavior [18]. Of course, it may be argued that including participants with limited previous experience could have provided different results. To ensure a correct understanding of a patient’s problem and selection of the optimal intervention, cognitive decision-making processes have been underlined as essential to achieve quality nursing [19]. Hwang [20] stated that nurses’ decision-making processes should be founded on evidence-based knowledge, as well as personal knowledge and experience gained in clinical settings. Specifically, nurses with higher levels of knowledge and clinical competency have been found to make clinical decisions more intuitively [21, 22]. Hence, shorter or no previous nursing experience prior to entry to the NA education program may impact on the students’ level of non-technical skills generally and in clinical decision-making specifically, throughout and at the end of the program. This again may lead to a need for revision of the curruculum, to ensure a certain level of non-technical skills before building this competence further due to removing the prerequisite. Until date, NA students in Norway have had the prerequisite of a minimum of two years clinical nursing practice prior to entry to the NA education program. Hence, this study should be repeated in students without clinical experience as a nurse both during and after completion of the NA, or other APN, education programs.

Additionally, participants in the current study reported having procedural and technical competence, as well as competence in medication administration, gained through their previous experience as nurses. This allowed for focusing on anesthesia specific issues in the NA education program, and that they felt able to ‘see the patient’ despite the high-technological environment. It would be interesting to explore whether lack of such experience is viewed as a challenge for inexperienced nurses partaking NA education programs. A key aspect of anesthesia practice is the ability to efficiently and safely perform practical procedures. Gaba et al. [23] differentiated between technical performance, as the ‘adequacy of actions taken from a medical and technical perspective’, and non-technical performance. According to Pedowitz and Marsh [24] there are three stages in the acquisition of procedural skills: cognition, integration, and automation. Cognition includes developing an understanding of the task. Integration means incorporating the knowledge from the cognition phase into the learning of the motor skills for the specific task. Finally, the task becomes automatic and even subconscious. The acquisition of competence in a procedural skill requires experience through a variable number of attempts depending on the skill [25, 26]. Without previous clinical nursing experience, students would need to progress through all these stages during the NA education program alongside learning anesthesia-specific tasks.

Some studies focusing on prerequisites to nursing education have been conducted. For example, Yousafzai & Jamil [27] reported a significant relationship between admission criteria and the academic performance of nursing students. Moreover, a 2020 scoping review found that academic program admission criteria, nonacademic program admission criteria, and admission criteria formulas or scoring systems were predictors of student program success [28]. However, the authors concluded that significant gaps in the literature exist regarding standards for determining program admission criteria that may be used to predict student success in undergraduate nursing programs. Another review suggested that it is challenging to isolate one single variable as the best predictor of student success, but that using a combination of variables can offer a reliable prediction method [29]. Yet, we have not been able to identify further studies than Burns [6] focusing on the specific prerequisite of clinical experience.

Benner [30] explained the acquisition of nursing expertise through five expertise levels: novice, advanced beginner, competent, proficient, and expert. Here, nurses at the novice level are still in nursing school. At the advanced beginner level, nurses use learned procedures and rules to determine what actions are required in a concrete situation. When competent, nurses are task-oriented and structure their work to achieve specific goals. At the proficient level, nurses perceive situations as a whole and have more ability to recognize and respond to changing circumstances, and finally expert nurses recognize unexpected situations and can alert others to potential problems before they occur [31]. Participants in our study may be compared to the proficient and competent level as nurses.

Nurse competence has been shown to increase with experience [32–34]. McHugh & Lake [35] stated that clinical nursing expertise is related to individual nurse level of education and year experience. Lima et al. [32] found a significant increase of the competence level in the first 6 months of transition from nursing students to Registered Nurses. Lejonqvist et al. [36] showed that therapeutic interventions require contextual awareness and co-ordination skills that develop during practice and use. When being exposed to the anesthesia setting, the participants in our study also acknowledged the lack of competence. However, being competent in some areas, transferable to the new setting, made the capacity to learn both new theory and new practical skills wider.

### Strengths and limitations

Within the qualitative design also lies the lack of generalizability of findings. It may be discussed that the trustworthiness of the findings in this study is challenged due to the limited number of participants, from one university and in a Norwegian setting only. However, participants came from four different hospitals, of both gender and a variation in age and years of experience as a nurse. Also, our interpretation was that we had reached saturation in our data, indicated by the identification of no new themes in consecutive interviews. Additionally, member checking throughout the interview was used to increase the credibility of the findings, and the agreement between participants also strengthen this. However, we could of course have presented the results to the participants as well.

Also, it may be seen as a limitation that the study was conducted as part of a masters’ degree, and by NAs with limited experience themselves. However, reflexivity was sought throughout the analysis and presentation of results, also involving the last and more experienced author.

## Conclusions

This study adds knowledge about the value of previous clinical nursing experience before entry to a NA education program- as experienced by NA students. Non-technical and technical nursing competence represented a solid base for achievement of anesthesia competence within the same areas, also ensuring patient-centred practice. The change in prerequisites to the NA education program must be followed by evaluations of consequences on students’ academic progress and competence at the end of the program, as well as a possible increased need for supervision throughout.

### Implications for further research

There is a need for ongoing evaluations of potential implications for future NA education programs, also following changes in prerequisites.

## Data Availability

The datasets supporting the conclusions of this article are included within the article.

## Abbreviations

APN: advanced practice nurse/nursing
NA: nurse anesthetist/nurse anesthesia
